# "Now we are in a different time; various bad diseases have come." understanding men's acceptability of male circumcision for HIV prevention in a moderate prevalence setting

**DOI:** 10.1186/1471-2458-12-67

**Published:** 2012-01-22

**Authors:** Angela Kelly, Martha Kupul, Lisa Fitzgerald, Herick Aeno, James Neo, Richard Naketrumb, Peter Siba, John M Kaldor, Andrew Vallely

**Affiliations:** 1 Sexual and Reproductive Health Unit, Papua New Guinea Institute of Medical Research, PO Box 60, Goroka EHP 441, Papua New Guinea; 2 International HIV Research Group, School of Public Health and Community Medicine, University of New South Wales, Sydney, Australia; 3 School of Population Health, University of Queensland, Brisbane, Australia; 4 Kirby Institute (formerly the National Centre in HIV Epidemiology and Clinical Research), University of New South Wales, Sydney, Australia

**Keywords:** Acceptability, Male circumcision, Papua New Guinea, HIV prevention

## Abstract

**Background:**

Adult male surgical circumcision (MC) has been shown to reduce HIV acquisition in men and is recommended by the WHO for inclusion in comprehensive national HIV prevention programs in high prevalence settings. Only limited research to date has been conducted in countries experiencing moderate burden epidemics, where the acceptability, operational feasibility and potential epidemiological impact of MC remain unclear.

**Methods:**

A multi-method qualitative research study was conducted at four sites in Papua New Guinea (PNG), with 24 focus group discussions and 65 in-depth interviews carried out among 276 men.

**Results:**

The majority of men were in favour of MC being introduced for HIV prevention in PNG and considered improved genital hygiene, enhanced sexual pleasure and culturally appropriateness key factors in the acceptability of a future intervention. A minority of men were against the introduction of MC, primarily due to concerns regarding sexual risk compensation and that the intervention went against prevailing cultural and religious beliefs.

**Conclusion:**

This is one of the first community-based MC acceptability studies conducted in a moderate prevalence setting outside of Africa. Research findings from this study suggest that a future MC program for HIV prevention would be widely accepted by men in PNG.

## Background

Increasing emphasis is being placed on biomedical technologies for HIV prevention, such as adult male surgical circumcision (MC)[1], vaginal microbicides [2], pre-exposure prophylaxis [3,4] and treatment as prevention [5]. Interest in MC has attracted unprecedented attention since it was shown in large-scale clinical trials in Africa to have a protective efficacy of around 60% in preventing HIV acquisition in heterosexual men [6-8], confirming earlier observational and ecological studies [9,10]. This led WHO and UNAIDS to recommend MC be considered an essential component of comprehensive HIV prevention in high prevalence settings [1]. Mathematical modelling by several independent groups has shown that MC, even with partial uptake, would be highly cost-effective and could avert up to 5.7 million new HIV infections and 3 million deaths over 20 years in sub-Saharan Africa alone [11-13]. The greatest impact of MC on HIV prevention is likely to be observed in communities where HIV rates are high and MC rates are low, such as in many countries in East and Southern Africa [13-15]. Targeting core groups of men at-risk is also likely to be highly effective in many settings [12,15]. These findings and the attempts to scale-up MC as a result have not been accepted into HIV discourses without debate, particularly among social scientists [16-18].

African research suggests that MC is generally regarded positively in both non-circumcising and circumcising populations [19-27]. Among non-circumcising populations, factors influencing the acceptability of male circumcision include improved genital hygiene; a reduction in HIV/STI and penile cancer risk; and low cost. Barriers to acceptability include cultural tradition; fear of pain, excessive bleeding; safety concerns; and high cost. In communities that practice traditional male circumcision, key incentives to continuing these practices appear to be a desire to maintain ethnic traditions; to enhance male sexual pleasure and performance; genital hygiene and aesthetic considerations. In many non-circumcising populations, women were more strongly in favour of MC than men, and among both sexes, the willingness to have male infants circumcised was greater than that of adult males to undergo circumcision themselves [22,23,25].

In a review of MC acceptability studies in sub-Saharan Africa, Westercamp and Bailey [25] found that among uncircumcised men, the median proportion willing to be circumcised was 65% (range 29-87%) across all studies, and 71% (50-90%) were willing to circumcise their sons. Among women, 69% (47-79%) and 81% (70-90%) thought circumcision was acceptable for their partners and sons respectively. Given the consistency across studies, the authors recommended that no further research on notional acceptability be conducted in Africa but that researchers and policy makers instead focus on pilot studies and phased intervention roll-out as part of comprehensive national HIV prevention strategies. Others have suggested a more cautious approach on the grounds that in many communities, complex cultural, ethical, medical and strategic issues need to be addressed before such interventions can be rolled out [26,28,29] For example, there are concerns that MC could displace pre-existing prevention measures such as condoms or behavioural risk reduction strategies [26,30], although a recent phase III intervention trial in Kenya observed no such behavioural risk compensation [7].

### Male circumcision and HIV in Papua New Guinea (PNG)

PNG reported the first person diagnosed with HIV in 1987. Since that time the available epidemiological and behavioural data have indicated the potential for a major HIV epidemic. Recent national adult prevalence estimates suggest the epidemic may be progressing less rapidly than previously feared with 0.9% of the adult population currently estimated to be infected [31]. National HIV prevalence however remains among the highest in the Asia-Pacific Region. Roughly equal numbers of male and female cases of HIV have been diagnosed in PNG, which is suggestive of a heterosexually driven epidemic. The epidemic exhibits substantial geographic heterogeneity, with cases clustered in a number of key provinces [32]. Innovative strategies for HIV prevention, treatment and care are urgently needed to address this complex public health issue in a country with unparalleled geographical, linguistic and cultural diversity [33,34]. Such tremendous diversity in a country experiencing a moderate-prevalance epidemic means that it is difficult to translate research findings from other contexts into public health policy for HIV prevention in PNG, and necessitates that country-specific research be conducted to guide future strategy [34].

There is limited published literature on MC in PNG and little contemporary information on penile cutting, associated penile practices and their socio-cultural context [35-37]. It is acknowledged in the available literature that MC is generally uncommon, unless carried out by a medical practitioner, and that most ethnic groups do not traditionally circumcise men [38,39]. A variety of penile cutting practices have been described in PNG, including different types of dorsal longitudinal foreskin slits [40], but only limited research conducted on the diversity of these practices, their historical and cultural context, derivation or meanings in terms of male initiation, sexuality and constructions of masculinity [35,37,41-43]. Many traditional penile cutting practices and initiation rituals appear to have been discontinued over the last few decades, but some appear to have persisted or been revived, adapted and re-interpreted [37,41,43]. Recently there has been acknowledgement of the spread of non-traditional types of penile cutting, penile inserts and other practices among men e.g. the insertion of ball bearings, beads and other objects into the skin of the penile shaft [37,39,44]. As discussed elsewhere [40,45], a greater in-depth understanding of these practices and their motivators is required in order to develop culturally nuanced HIV prevention policies and programs, especially in relation to MC.

Although research to date has appropriately focused on the role of MC for HIV prevention in African countries experiencing high-burden epidemics, the lack of research from moderate-burden settings (such as many countries in the Asia-Pacific region)[46-50], means that the acceptability, operational feasibility and potential epidemiological impact of MC remains unclear in these settings. Only four relevant studies have been conducted to date in countries experiencing low or moderate burden epidemics: an acceptability study among men at increased risk of HIV/STI acquisition in Thailand [48]; a clinic-based study among mothers of male children in India [46]; and research among men at increased risk of HIV/STIs in India [47] and Haiti [51]. In this paper we report findings from a community-based MC acceptability study and discuss the implications for future public health policy in PNG.

## Methods

**Figure 1:**
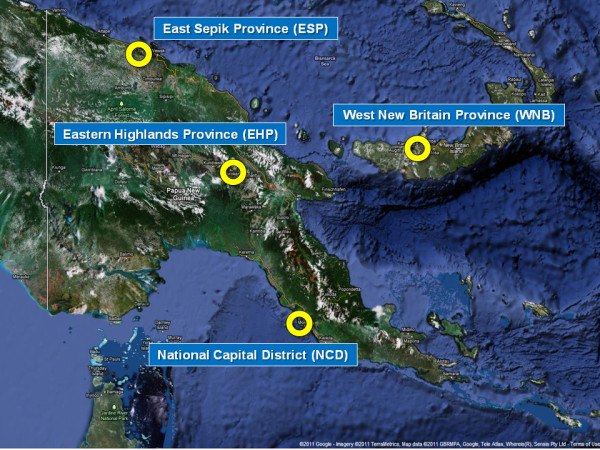
**Location of study sites**.

A multi-method qualitative study was undertaken in four provinces, each representing one of the four socially and geographically diverse regions of the country: National Capital District (NCD); Eastern Highlands Province (EHP); East Sepik Province (ESP); and West New Britain Province (WNB) (Figure 1). NCD was selected because it is the capital of PNG and has attracted in-migration from all regions of the country and beyond, creating a diversity of peoples and practices. EHP is considered a non-traditional penile cutting cultural area but is linked with major areas of the country through the Highlands Highway. ESP and WNB have anthropologically documented traditional penile cutting practices [36,41,52].

An iterative, purposive sampling technique was used to identify potential study participants following initial contact and interviews with key local stakeholders and community members at each study location. In order to speak of 'men' in the broadest sense a form of purposive sampling known as snowball sampling was used to gain access to a variety of PNG men rather than one group of them and this included hard to reach men such as those who had undergone a penile modification (traditional penile cut; contemporary cut; penile inserts; penile bloodletting; and MC [40]). Other men were recruited through towns, settlements, villages, schools, universities, workplaces (including National, Provincial & District AIDS Councils, and health care services/agencies), and church groups.

Men aged 16 years and over were invited to participate in the study and asked to complete written informed consent procedures in English or *Tok Pisin *(a lingua franca of PNG) prior to their enrolment. Participants were advised that they could withdraw from the study at any time without prejudice. A combination of focus group discussions (FGDs; each involving 6-9 participants) and in-depth interviews (IDIs) were conducted by age and gender-appropriate interviewers, all of whom were Papua New Guinean nationals with extensive prior experience and training in sexual health research. All interviews were digitally recorded, transcribed verbatim and where necessary translated from *Tok Pisin *to English at the PNG Institute of Medical Research (PNG IMR) in Goroka, where qualitative data coding and thematic analysis were conducted. All personal identifiers were removed from the interview transcripts and pseudonyms given to each participant.

Ethical approval was obtained from the PNG IMR Institutional Review Board, the Medical Research Advisory Committee in PNG and the Research Advisory Committee of the PNG National AIDS Council as well as from the Human Research Ethics Committees of the University of Queensland and the University of New South Wales in Australia. Approval from key local authorities, chiefs and other stakeholders was also obtained prior to the arrival of the research team at each location. Researchers conducted an interactive community meeting (*tok save*) at each site prior to the start of the study in that setting.

## Results

A total of 24 FGDs and 65 IDIs were conducted with 276 men across the four research sites (Table 1; Figure 1). The majority of men in the study were in favour of the PNG government implementing a MC program for HIV prevention among men. There were no differences in the reasons provided for or against the acceptability of MC between men who had previously undergone some form of penile modification and men who had not; and no differences in reported acceptability by geographical location. A small, but important group of educated men from Port Moresby who worked in the area of HIV prevention and care, accepted MC on the grounds of the potential health benefits to PNG men, but ultimately felt the introduction of MC would be ill advised due to the poor state of PNG's current health system and due to concerns regarding the risk of further consolidating male sexual dominance in the negotiation of heterosexual sex.

**Table 1:** Summary of FGDs and IDIs conducted, by study site and gender of participants

Participant Type	Study location								Gender			
	
	EHP		ESP		WNB		NCD		Male		Female	
	**FGD**	**IDI**	**FGD**	**IDI**	**FDG**	**IDI**	**FGD**	**IDI**	**FDG**	**IDI**	**FGD**	**IDI**

Community-based interviews with men who had a penile modification^1^	0	7	0	16	6	6	1	8	7	37	0	0

Community-based interviews with men without a penile modification^2^	5	0	4	0	2	1	7	3	14	4	4	0

Community-based interviews with women^3^	3	1	5	7	4	3	4	4	0	0	16	15

Key informant interviews^4^	0	3	1	8	2	7	1	9	3	24	1	3

Total FGDs and IDIs	8	11	10	31	14	17	13	24	24	65	21	18

**Total interviews**	**19**		**41**		**31**		**37**		**89**		**39**	

^1^Includes FGDs and IDIs with older men, younger men, mixed groups of men; and 13 IDIs with men carrying our traditional and contemporary penile practices

^2^Includes FGDs and IDIs with older men, younger men, mixed groups of men

^3^Includes FGDs and IDIs with older women, younger women, mixed groups of women; and 6 IDIs among female sex workers [*to be reported in a seprate paper by Kelly *et al.]

^4^Includes interviews with local chiefs, village elders, councillors, health care workers; representatives of National and Provincial Departments of Health; representatives of the National AIDS Council and from Provincial AIDS Committees

When interpreting these findings it is critically important to bear in mind that in PNG there is little to no distinction made between different forms of penile cuts. All penile cuts, irrespective of whether the foreskin is removed or not, are grouped together as 'circumcision'. This posed tremendous challenges to the conduct and interpretation of this study. In order to facilitate a more accurate discourse on circumcision in PNG, the authors of this paper have already developed a typology of penile practices [40,45]. Our research has indicated that only men who have undergone medical MC report full removal of the foreskin, and that this is rare in both traditional and contemporary penile practices, where dorsal longitudinal slit is the most common penile cut performed. However, most men with a dorsal slit refer to their own cuts as 'circumcision' whilst *kutim kok *is typically used to refer to all types of cuts in *Tok Pisin*. It is unclear whether they consider such cuts to confer protection against HIV acquisition: our earlier research suggests that enhanced sexual pleasure, promotion of penile hygiene and prevention of sexually transmitted infections are the key motivators for these practices [43,45]. A further complication is that dorsal longitudinal slit of the foreskin typically results in the remnant foreskin retracting from the glans resulting superficially in an appearance similar to complete foreskin removal [40]. Thus, many respondents talk of having had a dorsal slit and then being 'foreskinless' when in fact no skin has actually been excised (e.g. as stated by respondents such as Pierson and Naldo, below). Additionally, in order for men to form an opinion regarding the acceptability of MC for HIV prevention, researchers in the majority of cases, needed to provide a basic overview of the research evidence to date from clinical trials in Africa (e.g. MC provides men with partial protection of around 60% during vaginal sex with a woman; MC does not protect women from being infected from an HIV-positive circumcised male).

### Male circumcision is an acceptable intervention for HIV prevention in men in PNG

Men provided a number of key reasons for their acceptance of MC and these were, in order of emphasis: that MC will prevent HIV and STIs; MC would improve penile health and hygiene; MC is a culturally appropriate practice and; MC would increase sexual pleasure (Table 2).

**Table 2:** Acceptability of MC for HIV prevention among men in Papua New Guinea

Reasons why MC considered acceptable	Reasons why MC considered unacceptable
• *MC prevents HIV and STIs*	• *MC would promote sexual risk compensation*

• *MC improves penile health and hygiene*	• *MC is culturally inappropriate*

• *MC is culturally appropriate*	• *MC goes against religious beliefs*

• *MC increases sexual pleasure*	• *There is not enough PNG-specific research evidence*

	• *There are other, more effective methods*

	• *MC is a gender-biased prevention*

	• *MC would be a burden on the PNG health system*

#### MC prevents HIV and STIs

The majority of respondents considered MC an acceptable intervention for HIV prevention, based on the information provided by the research team in regards proven efficacy in Africa. Some men believed that MC would also confer protection against STIs, which further enhanced their view that a future intervention would be acceptable to them and to other men in PNG, as typified in the following remark: 'It's safe for the HIV/AIDS virus not to be transmitted when you sleep with a woman' (Elmo, ESP).

Introducing MC and advocating for the simultaneous use of condoms would allow for what many of the men referred to as 'double protection': 'The way I analyse is it will be like double protection, using condom and you circumcising and using condom together.' (Rodam, ESP). By combining HIV prevention methods, 'it can help us to prevent this [HIV] more effectively' (Kano, ESP); 'Both of them must work together' (Dennet, EHP). In addition to reducing personal risk of acquiring HIV, men also described community and population-based impacts of MC:

When circumcision is implemented and when all the men do that... the number of HIV/AIDS cases will be reduced and we won't see many people and many children whose parents have died ...Our businesses would run properly...and we would see our country change and develop..We will see families with money, children will go to school and there will be food in the house because men and women will have the strength to create gardens. (Augustine, EHP)

Conflating MC with dorsal slits (both referred to as circumcision--*kutim kok*), some men who had undergone a dorsal slit believed that the protective factor of MC for HIV (and STI) prevention was also available to them. Bolton, who resides just outside of the NCD, had undergone a contemporary penile dorsal slit in the past and carries out this procedure on his peers, stated:

Circumcision from my experience I see that it's a bit all right... If they [young men and upcoming generation] circumcise to prevent AIDS or sexually transmitted diseases such as gonorrhoea, if you circumcise you won't get these diseases as well. (Bolton, NCD)

Providing further cultural narrative of the conflation of MC with dorsal slits, Pierson shared his views:

From those who were circumcised they used to tell us that. 'In the past when we were with foreskin and sleep around with women, we usually get sick frequently. After we've circumcised and now that we are foreskinless we do not get sick.' When they said that we found it really hard to cut this, where will we get the treatment or razor blades and all these. (Pierson, EHP)

Having heard that MC could prevent HIV acquisition a number of the males who had a contemporary dorsal slit reported that they had done so in the belief that their 'circumcision' would provide them with protection: "I didn't want to get the bad disease [HIV] that's why I removed my foreskin.' (Naldo, EHP). Naldo stated that he had removed his foreskin but like Pierson, had in fact undergone a dorsal slit, and therefore his foreskin still remained but had retracted to the sides of the penile shaft, resulting in a superficial appearance consistent with complete removal. Furthermore, many men advocated to others that they too must undergo penile cutting in order to prevent themselves from being infected with HIV or STIs:

In the community I keep on telling the boys like this: 'Listen, you must cut your foreskin. Now we are in a different time, various bad diseases have come...if you cut your foreskin you will remain well. You will remain as a man, not a single disease will find you. (Henriot, ESP).

Having already cut their foreskins, many of men who had undergone a contemporary cut, particularly from Eastern Highlands Province, felt more confident to have sex with an HIV-positive woman having heard that MC conferred protection against HIV infection.: 'I feel that I've already been circumcised so this sickness [HIV] like whatever sickness they [women] acquire, when I have sex with them I will not get it' (Cornelius, EHP).

Although it was clearly HIV and STI prevention in men that respondents saw as key benefits of MC, another albeit smaller subset of men saw that women could also benefit from MC, particularly in relation to cervical cancer, or what they describe in *Tok Pisin *as *sik bilong mama*:

[MC means] avoiding the chances in acquiring these diseases usually being transmitted through sexual means, like gonorrhoea, syphilis, donovanosis, HIV/AIDS and cancer of the cervix. Diabetes also comes with them. (Zachariah, ESP).

...the mothers get the illness of the womb, it is related to such dirt sticking to the man's penis and sometimes the men usually come [home] drunk, they don't think about washing, they come throw whatever and go [have sex]. (Sacha, ESP)

ESP is the only province in the country implementing a program, albeit in an ad hoc fashion, of MC for HIV prevention. Community awareness started in October 2006 prior to program implementation in December 2006. In the three-year period between the start of program implementation and this study, the MC program coordinator in ESP reported that a total of 490 men had undergone adult male surgical circumcision. According to the coordinator, prior to the procedure being carried out through mobile clinics in remote areas, both men and women in the communities are informed of the benefits of MC in that it prevents HIV and STIs (specifically gonorrhoea, syphilis and donovanosis) in both men and women, and cervical cancer in women. With this in mind, it is not surprising that men from ESP identified more than any other groups of men, the benefits of MC in reducing both HIV and STI transmission:

I've been to the hospital, you know about HIV, there is more than just HIV, different types of sexually transmitted infections are also there. That's why I thought about this and I went and got myself circumcised at the hospital. (Hadrian, ESP).

#### MC improves penile health and hygiene

Penile health and hygiene was the second most common reason for men supporting the implementation of MC for HIV prevention in PNG. The primary perceived hygiene benefit of MC was that it would result in a *drai *(dry) and *klin *(clean) penis, absent of *doti *(dirt), *gris *(grease) and *smel nogut *(bad smell).

Okay,...this foreskin of the penis, when you leave it for two or three days, you pull it down and you have a look, lots of dirt, this dirt will be inside...Stuff like grease and other things like this will be under the foreskin on the penis. It will smell terribly. It's good to cut the penis in order to remove the dirt. If you leave it exposed the penis will remain clean. (Fredrick, ESP)

From my understanding, a man won't get HIV/AIDS because all the dirt after he has finished having sex and ejaculates, no germs will stay on the penis. When the skin is there, dirt remains. When the foreskin is removed the penis will be clean. (Fabian, NCD)

Drawing on traditional constructions of masculinity, male health and strength as a result of bloodletting (albeit from penile cutting or from urethral bleeding) several participants said the following of MC:

A man who is not fat, a skinny man; when you circumcise his penis, he will fatten ...and some full grown boys, they usually grow slowly slowly so when you cut their foreskins you will see that they grow up very quickly. (Aquinas, ESP)

Building on this belief in health and hygiene, the men from traditional bloodletting communities, particularly in ESP, linked MC and the benefits it offers to those conferred by penile bloodletting practices. Both MC and bloodletting practices are for men; both result in improved physical and penile health; and both result in the release of 'bad' maternal blood, albeit in different ways. For men with penile modification, especially those from traditional circumcising communities, keeping their penises clean and healthy was central to why they had undergone either a traditional or contemporary penile modification. Therefore MC was seen as reinforcing their concerns about penile hygiene, albeit it in a more medicalised setting. This concern with hygiene in the traditional setting was not solely the concern of men, as Korowa, a Chief from WNB said of women's concern about penile hygiene:

...our old women, they usually say that you must cut the foreskin of your penis, it would be clean and dry. Like you know when the boys usually pull the foreskin of their penises back they have this grease, the white stuff remaining there, these are the things that our mothers usually say are unhealthy. ...Sometimes like it will smell or it has got sickness, they usually tell us little boys. (Korowa, WNB)

Highlighting the sociocultural relationship between beliefs about how MC prevents HIV and STIs and the role of penile cutting in promoting penile hygiene, a large number of the men conflated the two reasons for accepting MC:

From my point of view I analyse that circumcision is best because it will prevent us from getting HIV/AIDS. ...from some awareness I attended too I heard they said that if there is skin still remaining and when you have sex with a woman, those grease of woman will be attached if the woman has the disease, HIV/AIDS will be stuck within the grease and it will stick to your skin and remain there and it will be easy to go into the hole of the penis and go give sick.(Alexis, NCD)

#### MC is culturally appropriate

Taking culture to refer to shared attitudes, goals and practices, MC was considered acceptable in both traditional and contemporary cultural contexts.

Men from traditional penile cutting communities were by and large supportive of MC, many because they believed that their traditional cut, albeit somewhat different from MC, was amendable to a hospital setting cut. That is, they would support their sons to have MC in a hospital if they were then permitted to carry out their traditional practices back in the village men's house. Odilo from WNB, but now living in NCD said:

If the government permits circumcision maybe for us coming from this custom with circumcision, we won't find it hard because we are already in it because it is our custom. But for men who do not come from this kind of custom maybe they will find it a little bit hard. But as I mentioned already for us people who come from this background, we would only agree for our children to go and circumcise, and then we can go back and do our customs. (Odile, NCD)

Other men were more reserved and offered cautionary acceptance. These men foresaw that the skills and personnel to carry out MC were already present in the community where traditional penile cutting was undertaken: '...it's our traditional custom from the past to today. So there is no need for us to go to the hospital.' (Aubert, WNB). Some men suggested that local cutters be trained to carry out full foreskin removal, especially in the area of medical treatment for post-operative care.

This is like it our custom and you said that if the government makes a law we will support it but it is much better for the government to come back again like give us medicines to help us men who cut foreskin and we will cut it. So they can provide employment back to us to circumcise like this will not affect our custom. (Moritz, WNB)

Other participants suggested that male health care workers could participate in traditional customary rituals by providing MC in the men's' house so that the cut would occur in the appropriate socio-cultural context. Several traditionally circumcised participants from Madang and Manus (now residing in NCD) reasoned that MC must be done in a traditional men's' house to achieve the full behavioural change that accompanies initiation because within such a structure, regulations exist in order to control and determine appropriate masculine (and therefore sexual) behaviour:

If the government encourages or enforces this as a country-wide practice or, I still support my brother over there because within a traditional setting ... we have strings attached. There are beliefs and there are disciplines, rules or code of ethics of our traditional practices. So once you go into a men's house you are attached, I mean after you circumcise they have you there with talks and rules and regulations that you follow to control your behaviour and attitude. (Elias, NCD)

Part of the structure, which allows for the regulation of men's behaviour, is that the penile cut and associated rituals are witnessed by the leaders and the community, and furthermore, it is important to the maintenance of the clan's identity, as shared by Irvin from WNB:

I have [such] strong belief in my custom that my son... . I will take him back again to the village to perform this custom so the leaders from the village will know that he is my son and he is part of this clan. I must bring him back so whatever discussions in the future, the people from the village know that this child is from this place too and he is part of this clan. (Irwin, WNB)

Reflecting on the decline in traditional penile cutting practices in many areas of PNG, one of the traditional chief's in WNB whose community continues to initiate boys as young as 5 years, says of accepting MC for HIV prevention, that the PNG government has a responsibility to both advocate for and support the continuation of traditional penile cutting practices as part of men's initiation. He has witnessed that with development 'this practice [of penile cutting] has died away'.

While MC was largely acceptable to traditional circumcising communities, several men from these communities cautioned that their acceptance and the government's desire to implement MC should not mean that MC becomes mandatory for all Papua New Guinean men, particularly those who originate from non-traditional penile cutting communities.

As we have argued previously [43], traditional and contemporary penile practices share similar socio-cultural meanings with evidence of appropriation observed between traditional and contemporary penile practices. With the appropriation of traditional penile modification practices such as penile cutting and bloodletting ('shooting') by young men, a new culture of penile modification has been cemented into the cultural landscape of PNG. This appropriation provided an additional cultural context within which MC for HIV prevention was perceived as acceptable. This was especially evident in what can be best described as 'prison culture' [39,40,53].

Men from communities where traditional penile and/or urethral bloodletting practices have died out, and who have undergone contemporary penile cutting in the absence of traditional customary observances, thought that MC was culturally acceptable: '...with regards to circumcision too, like when I talked about custom [of bloodletting] it is similar as well'. In fact, many suggested that MC resulted in the same sociocultural and medical outcomes of traditional penile cutting and bloodletting practices: manhood, strength and the removal of maternal blood. Others however suggested that along with MC, the penis should undergo separate but simultaneous bleeding.

#### MC increases sexual pleasure

Perceived improvement in sexual pleasure for men and their female partners was another common reason why men considered MC acceptable for HIV prevention:

... the penis without a foreskin when you go to have sex with women, like you feel good. It is good when the penis enters really well and when you release it's much better. (Aaron, ESP)

Alright if you want to satisfy woman and woman feels it probably she gets...sexual satisfaction. She feels that 'oh yeah, you removed foreskin, yeah, you are coming good so she will feel happy with this kind. (Ilanus, NCD)

The notion that MC could increase sexual pleasure was reported by all men, with and without a penile modification, but primarily championed by those men who had an existing contemporary penile cut. Men who had undergone a traditional or contemporary penile cut or MC, reported that circumcised penises became 'extra thick and extra long', and that cutting results in 'prolonged erection' thus allowing the men to go 'round after round'. Increased pleasure was also achieved by the absence of both the foreskin and a condom so that sexual intercourse was truly 'skin-to-skin':

I used to think 'leave condom use, I have not gained any pleasure from condoms'. If I used the 'bald man' [circumcised penis] plainly my bald man will really test you. When I use the bald man and we have direct sex skin-to-skin, then the woman usually gets the real feeling. (Henriot, ESP)

### Male circumcision is unacceptable for HIV prevention in PNG

A minority of men from across all study sites said that they would not support the introduction of MC for HIV prevention. The primary reason provided was that MC would have a negative overall effect by promoting sexual risk compensation among men. Others were concerned that MC would be culturally-inappropriate and go against prevailing religious beliefs (Table 2).

#### MC would promote sexual risk compensation

Men who were against the introduction of MC in PNG almost exclusively objected because they feared men would develop what they called a 'false sense of security'. While this is how men phrased their concerns about possible behavioural changes post circumcision, men's sense of security is not accurately best described as false because circumcision does reduce risk, although not totally. Risk compensation is used in the literature to describe the notion that individuals modify their behaviour in response to real or perceived changes in risk [54]. Men described what has been defined as 'sexual risk compensation' whereby the likelihood of men continuing with or adopting measures associated with lower HIV acquisition risk, such as partner reduction or condom use, are reduced. Respondents were concerned that men who undergo MC might perceive themselves to be totally protected from HIV risk post-circumcision. Participants felt that if men perceive MC to offer complete protection from HIV, there is a risk that condom use would decrease. Many men spoke of the new (absent) position that condoms would have in men's sexual relationships post-MC.

If it's [circumcision] marketed as a way to prevent you from contracting AIDS, people might get the false impression: 'if I am circumcised, no matter how many times I go out with how many different women, I'll be ok'. And when they are under the false impression you know that the risks of contracting the disease are far higher. (Percy, NCD)

Okay with that now it will be like the usage of condom they will say, 'Ha, I am circumcised already so there's no need for me to use condom and with that now condom [use] will drop. (Nelson, NCD)

Providing anecdotal evidence of the fears expressed in the above quotes, male health care workers reported that many men who were circumcised returned with STIs because they did not use a condom, thinking circumcision protected them.

Most of the guys I've talked to, and especially those who came with STDs, they have been telling me that they've been circumcised already and they think that they are safe, so most of the time they don't use condoms. (Dr. Mal, WNB)

The other big problem is that those of us who have been involved for a long time in sexual health issues in PNG are very concerned about the idea that 'I'm circumcised already so I won't get it now. Forget about using condoms.' (Dr. Bill, NCD)

Many participants who felt MC was ill advised for PNG pointed out that removing the foreskin would stimulate men to increase their sexual activity due to increased sexual desire, leading men to increase their number of sexual partners and potentially promoting physical and sexual violence against women, including their wives and regular sexual partners. Young men in NCD were concerned that a *kela kok *(circumcised [literally 'bald'] penis) would result in men being more easily and frequently aroused, for example when the penis rubs against their trousers, or when a men sees a woman 'dressing up nicely'.

Okay from my experience is when you still have your foreskin, your sexual arousement is not that high. When I am circumcised, and if you see a woman not walking properly or dressing up nicely or if she is sitting down wrongly or you used to think of her, then quickly your penis will be erecting. You won't control it... it automatically it will aroused. So every time those of us who are circumcised have gone through this experience, and we wore pants to avoid this...and once if you are wearing the trousers without your pants and the penis touches the trousers, even if there is no woman walking or you see them, you'll be aroused. So that's one disadvantage that I see, from circumcision. It will now depend on your individual control. Like if this is our tradition than you follow it. And if it isn't your tradition don't try it because if you tried it you will feel something different that you've never experienced it before will happen to you. And if it happens to you and you cannot control it, that's when troubles occurs from this kind... you will feel differently and sometimes you cannot control your brain, you will rape [her] on the spot. (Jarret, NCD)

Like just from my personal experience, when they cut my foreskin, I feel like having sex every now and then, but my wife used to dislike it. But now I go to the extreme... I hit her and all this. It's not good. And if we are going to say, 'okay now, everyone will be circumcised', we are going to create more problems again because the men will want to have [more] sex. (Barry, EHP)

#### MC is culturally inappropriate

For a small group of men, MC was considered against customary tradition because they thought the purpose of MC was not in alignment with the purpose of a traditional penile cut. These men were concerned that introducing MC into traditional penile cutting communities would result in the cultural meanings associated with penile cutting being distorted:

Mainly when they go to the 'haus tambaran' [spirit house] for initiation, circumcision usually happens too ... they go through to obtain the strength to hunt for meat, build a house, make a big garden. Not in regards to sex or such... lately now we are using circumcision like we are talking about the sexual side. (Galen, NCD)

Not only is the meaning different but an essential aspect to traditional penile cutting--the shedding of 'bad' maternal blood--would be lost and tradition interfered with:

Yes it looks like, the behaviour of penile cutting has increased and I will also be concerned because my elders have not being circumcised... yet, they cut it in the white man's way, just cutting the skin, pull and remove only, they did not shoot the blood. They did not cut everything and the blood did not come down, it's like that and they just cut and did not remove the blood yet. If it [MC] is implemented I will not be circumcised. I will just shoot the penis and cut and remove so the blood will come down. (Nicholas, ESP)

In penile bloodletting communities the issue was that MC was a one-off event while penile bloodletting was a continuous practice that enabled men to maintain their health and to prevent disease:

I think many of them will be a bit scared to remove their foreskin and many of them, if we teach them properly the way of using condom, I think they will go for the condom. Why will I have this thing of penile cutting and all this because I already went into my custom [of penile bloodletting] in the *haus boy*? So from my initiation [I know] that when I have sickness, it's something I can still do myself [penile bloodletting] to protect myself... to prevent sickness. (Sailor, ESP)

For others, MC was culturally unacceptable because in these men's communities there was no tradition of penile cutting or bloodletting:

In my place, it is a taboo. I mean Western Highlands, Southern Highlands both areas, it is forbidden to cut this kind of thing. I mean from our custom we don't have this kind of thing [circumcision]. (Hubert, NCD)

#### MC goes against religious beliefs

Although the specific Christian denomination of each respondent was not sought, as a Christian nation, it is not surprising that some participants considered MC unacceptable for religious reasons. For some men HIV prevention was to be found in God, and specifically, by observing a faithful Christian life and by respecting God's creation:

With regards to circumcision maybe we remain like this because when the Lord created us, he didn't talk about us circumcising or cutting or shaping our skin here and there. He said no. How He shaped us, we must remain like this. So those of us who have cut it, it is one thing that wouldn't look good and we must stop ourselves, we shouldn't. Marry only one person and stay. It is not to go and become promiscuous. That's where we will get [HIV]. So from my opinion, it is no to circumcision and no to promiscuity. (Jaspar, NCD).

## Discussion

The majority of men who participated in this study were overwhelmingly supportive of the introduction of MC for HIV prevention in heterosexual men in PNG. As observed in earlier research from East and Southern Africa [19-26], notional acceptability was high irrespective of whether men came from communities that practice traditional penile cutting or not; whether respondents had undergone any form or penile modification or not; irrespective of where men lived and the site of recruitment. Determinants of acceptability also echoed those reported in these earlier studies, such as perceived reduction in HIV acquisition risk; improved genital hygiene; and enhanced sexual pleasure. It is unfortunately not possible to make comparisions between our study findings and those from other countries experiencing moderate epidemics, or to compare our findings with other Asia-Pacific countries, due to the paucity of research conducted to date on MC in these settings [46,48].

A minority of participants objected to the introduction of MC, primarily due to concerns about sexual risk compensation, stating that men would change their sexual behaviours due to a 'false sense of security', resulting in increased sexual activity and numbers of sexual partners, and decreased condom use. Research from a variety of settings in Africa however, has found evidence of risk compensation among only a minority of men following MC [8,11,54-59] Recent evidence from Orange Farm in South Africa indicates that MC roll-out can have a significant impact on HIV transmission at community level [11]. This suggests that concerns about the possible impact of risk compensation post-MC may have been overstated, and that risk compensation can be mitigated by the delivery of locally-appropriate comprehensive and integrated HIV prevention programs including HIV risk reduction counselling session [57].

Some participants considered MC inappropriate for other reasons, primarily because they felt it culturally and religiously inappropriate. Culture has been increasingly acknowledged in both the spread and prevention of HIV. In particular, emphasis has been placed on developing programs and policies that are culturally sensitive and which build on the strengths of culture rather than destroy it. Nowhere has the issue of culture been more heated in relation to biomedical prevention than in the discourses on MC, with key social researchers emphasising the importance of addressing the cultural implications of any potential roll-out [17,18,29,60]. While the majority of men in our study identified culture as a reason for accepting MC, a minority cited culture in their objection to MC. Both traditional and contemporary cutting practices, and the differing emphasis that they place on constructions of masculinity and male sexuality in PNG, need to be addressed in any possible future MC program in this setting.

Religious reasons for non-acceptance were reported by a small number of men. However, because Christianity is 'as fundamental to Papua New Guineans way of understanding themselves as are their customary ways'[61], it is important that future policy discussions on MC address these concerns carefully. Having a 'faithful Christian life' was described by some participants as providing HIV prevention thereby making MC for HIV prevention redundant. There are many different Christian denominations and churches in PNG with diverse attitudes to HIV. Kelly [61] has outlined the important role of some denominations in responding to the HIV epidemic in PNG. Understanding how churches and religious organisations influence in-country discourses on HIV prevention in PNG, and working in partnership with faith-based organisations in the design and implementation of future HIV prevention programs, will be critical in future program success, should PNG decide to go ahead with a national MC program for HIV prevention.

In addition to the evidence that this study provides on acceptability it also highlights two important issues requiring careful consideration in PNG. First, the majority of men in this study had limited knowledge and understanding about HIV and STIs, particularly in relation to how infections are acquired, and the role of penile hygiene in their transmission. For example, many men believed that by cutting or removing the foreskin, the 'dirt' and 'white grease' that builds up under the forskin, from which they believed STIs and HIV are created, is removed. Second, given that *katim kok *or *katim skin *are used to categorise all forms of penile cutting in PNG irrespective of whether the foreskin is removed or not, future behaviour change communication strategies will need to clearly differentiate MC from other forms of penile cutting in this setting. In particular, the proven benefits of MC compared to other forms of penile cutting will need to be clearly articulated.

A challenge for HIV prevention in countries such as PNG that have great geographic, linguistic and cultural diversity is to develop culturally-nuanced and innovative policies and programs for HIV prevention. This paper has shown that men from a variety of different settings in PNG are notionally supportive of MC for HIV prevention which is encouraging, however it is important to recognise the ambiguities which surround *katim kok*, and the influence that local-level tradition and contemporary customs may ultimately have on the acceptability and uptake of a future intervention. We agree with Montgomery et al. [62] who state the need to re-conceptualise acceptability, focusing on people's experiences within specific cultural contexts, examining local meanings and understandings. We have attempted to do this and have illustrated how men are making sense of *katim kok *within a dynamic social and cultural context. As Montgomery et al. [62] state, 'local cultures are not static and unchanging, waiting to be studied and mapped by researchers, but dynamic and continually adapting to new circumstances and influences'.

As in other countries experiencing moderate burden HIV epidemics, a key challenge having investigated notional acceptability and the determinants of acceptability will be to decide how future interventions could best be implemented in order to ensure the greatest epidemiological and public health impact. Comprehensive guidance on these issues for low and moderate burden settings is not currently available but is urgently required to guide HIV prevention policy outside Africa. Complementary health systems research [40,63,64] and mathematical epidemic prediction modelling [65-67] being conducted by our group are assisting policy makers and development partners in PNG to negotiate these issues, and to develop policy based on robust scientific evidence. This approach may represent a model for other moderate burden countries considering implementing MC for HIV prevention.

## Conclusions

Research findings from this study suggest that a future MC program for HIV prevention would be widely accepted by men in PNG. Complementary research to establish the potential epidemiological impact, operational feasibility and cost-effectiveness of different MC implementation models are required to inform future public health policy in this moderate prevalence setting.

## End notes

^1^These sites were selected during a National Stakeholders Workshop held in Port Moresby in May 2008, attended by in-country government and non-government stakeholders and researchers from the PNG IMR and Australia-based research team.

^2^In each province the research team conducted research amongst multiple cultural and language groups, therefore we do not provide the anthropological details of each.

## Conflict of interests

The authors declare that they have no competing interests.

## Authors' contributions

AK, MK and AV conceived, designed and coordinated the study, conducted the literature review and drafted the manuscript.

LF, PS and JK participated in the design and coordination of the study and helped draft the manuscript.

MK, HE, JN, RN participated in the design and coordination of the study, conducted in-depth interviews and focus group discussions, and helped draft the manuscript.

All authors have read and approved the final manuscript.

## Pre-publication history

The pre-publication history for this paper can be accessed here:

http://www.biomedcentral.com/1471-2458/12/67/prepub
